# The multidimensionality of Japanese kanji abilities

**DOI:** 10.1038/s41598-020-59852-0

**Published:** 2020-02-20

**Authors:** Sadao Otsuka, Toshiya Murai

**Affiliations:** Department of Psychiatry, Graduate School of Medicine, Kyoto University, 54 Shogoin-kawahara-cho, Sakyo-ku, Kyoto, 606-8507 Japan

**Keywords:** Cognitive ageing, Human behaviour

## Abstract

The aim of this study was to identify the cognitive structures of kanji abilities in the Japanese general population and to examine age and cohort effects on them. From a large database of the most popular kanji exam in Japan, we analyzed high school graduation level data of 33,659 people in 2006 and 16,971 people in 2016. Confirmatory factor analyses validated the three-dimensional model of kanji abilities, including factors of reading, writing and semantic comprehension. Furthermore, the age effect on writing, and correlations between writing and semantic dimensions, were different between 2006 and 2016, suggesting reduced writing ability and stagnation in integrated mastery of kanji orthography and semantics in current-day Japanese adults. These findings provide the first evidence of the multidimensional nature of Japanese kanji abilities, and age/cohort differences in that dimensional structure. The importance of the habit of handwriting for literacy acquisition is discussed.

## Introduction

Writing systems can be broadly divided into phonographic and logographic scripts. In the former, a letter is mapped onto a sound unit, as in English. In the latter, a character is mapped onto a meaning unit, as for Chinese. Japanese uses both systems in combination, namely kana and kanji. Each kana letter represents one mora, a sub-syllabic unit of sound in Japanese, with highly regular and consistent letter-sound correspondence. In contrast, kanji characters usually have multiple pronunciations, which can also be written with more than one kana letter. The correct pronunciations of Japanese kanji words are determined by context and at the whole-word level, in a similar manner to English exception words, unlike the Chinese logographic writing systems. Kanji characters are used for content words (i.e. most nouns, or the roots of most verbs, adjectives, or adverbs), whereas kana letters are mainly used for inflectional endings, postpositions, or conjunctions. A single kanji character or more than two in the so-called compound words, occasionally accompanied by kana suffixes, can represent a word. Many homophones in the Japanese language are represented with the exact same kana letters but have different meanings, and can be discriminated by writing in kanji. In addition, unlike kana or alphabetical letters, kanji characters vary in visual complexity from simple characters such as the kanji カ (*chi-ka-ra* or *ryo-ku* or *ri-ki*, power) to very complex ones like 鬱 (*u-tsu*, depression), both of which are designated as daily-use kanji by the Japanese government.

Considering the unique properties of kanji, including multiple pronunciations, semantic values, and variability in visual complexity, it is assumed that the abilities to master and manage Japanese kanji would be multidimensional as for overall language ability^1,2^. However, the dimensional structure, whether uni- or multidimensional, has not been established. Given the higher prevalence of problems in literacy acquisition in kanji than in kana among Japanese children^3^, understanding the functional components of Japanese kanji abilities is practically important for reinforcing therapeutic and educational strategies for these problems. This study investigated the cognitive structures of literacy skills required to manage Japanese kanji.

When considering the dimensionality of Japanese kanji abilities, knowledge about acquired alexia/agraphia would be informative. Studies of Japanese patients with brain damage showed that neural underpinnings of reading, writing, and semantic processing of kanji differ substantially from each other^4^. Theoretical frameworks for explaining the written language performance in individuals with reading and/or writing difficulties have been provided by dual-route models^5–7^. Dual-route models hypothesize cognitive structures of the information-processing system for written language by two distinctive but interactive procedures. One is the lexical route for all familiar words at the whole-word level, and the other is the non-lexical route for unfamiliar words or non-words at the sub-word level. Whereas reading/writing by the non-lexical route relies on grapheme-to-phoneme or phoneme-to-grapheme conversion based on letter-sound correspondence, word-level reading/writing by the lexical route employs word-specific phonological and orthographic memory representations, and the corresponding conceptual representations in the semantic system^6^. According to these hypothetical models, literacy skills to manage Japanese kanji characters, each of which is a word or morpheme, depend on phonological and orthographic lexicons and the lexico-semantic system in the lexical route^6^. This supposition could be supported by the Japanese version of dual-route models^4,8,9^. In these anatomically-based models, different brain regions in the lexical or ventral route are proposed for storage of phonological, orthographic, and semantic information about kanji words^8,9^. Reading, writing, and semantic comprehension could thus be suggested as likely components of Japanese kanji abilities, distinct from each other but also interacting.

Clarification of dimensionality in kanji abilities would be beneficial for understanding not only individual differences in Japanese language skills, but also developmental changes in the abilities and effects of environmental changes on them. Kanji characters are generally acquired based on school grade everywhere in Japan, in strict accordance with the school curriculum guidelines developed by the Ministry of Education, Culture, Sports, Science and Technology^10,11^. In the first year of elementary school, children learn 80 characters of daily-use kanji (from a total of 2,136), after acquiring all 71 kana letters, with which the Japanese language can be written exclusively. They learn 160 new kanji characters in the second grade, 200 in the third grade, 200 in the fourth grade, 185 in the fifth grade, and are expected to master 1,006 kanji characters by the end of the sixth grade^10^. Then, they gradually learn to read all daily-use kanji and write most of them before their high school graduation^11^. More than 10,000 kanji characters and 50,000 compound words are included in general kanji dictionaries. Thus, Japanese kanji abilities continue to develop through adulthood. The curriculum guidelines for kanji education, which are the major constraint for the age of acquisition of kanji characters^12^, have not changed since 1989^13,14^, and then only 10 kanji characters were added to the characters to be learned in elementary education by the revision that year^15^. However, the rapid spread of digital writing devices, such as PCs and smartphones in recent decades, has drastically reduced frequency of handwriting, e.g., an 11.4% decrease in Japanese adults who habitually write letters by hand over the eight-year period from 2004 to 2012^16^. This is despite the fact that the frequency of kanji use (e.g., typing) does not seem to have changed. In addition, there was a 22.8% decrease in the number of Japanese people who took the most popular kanji exam in Japan, the Japan Kanji Aptitude Test (*Nihon Kanji Noryoku Kentei*: Kanken) over the ten-year period from 2006 to 2016 (about 2.6 million people in 2006, and about 2 million people in 2016)^17^. On the other hand, the number of people who took the Test of English for International Communication (TOEIC) increased by 63.8% over the same period (about 1.5 million people in 2006, and about 2.5 million people in 2016)^18^. Although opinion polls taken by the Agency for Cultural Affairs in Japan reported that public interest in learning the Japanese language, which includes spoken and written language, has not generally changed in recent decades^19^, increased attention to learning English or internationalization may have resulted in a decrease in the time and effort dedicated to learning Japanese kanji. These environmental changes possibly affect age-dependent acquisition of kanji abilities in Japanese, particularly the dimension related to writing accuracy or orthographic lexicon in adults, as well as integrated mastery of multidimensional kanji skills.

The primary purpose of the present study was to identify the cognitive structures of kanji abilities in the general Japanese population. To examine the validity of multidimensional models of Japanese kanji abilities, we retrospectively investigated a large database of the Kanken, using confirmatory factor analyses (CFA). In addition, we examined the effects of age and cohort on kanji abilities, using comparable data from 2006 and 2016. We hypothesized that (1) the three-factor model of Japanese kanji abilities, including factors of reading accuracy (kanji phonology), writing accuracy (kanji orthography), and semantic comprehension (kanji semantics), fits better than two- or single-factor models, (2) age of examinees affects kanji abilities factor-specifically, and in terms of the relationships among the factors, and (3) the pattern of age effects shown in 2006 data differs from that of 2016.

## Methods

### Nature of data

We investigated a large database of the most popular kanji exam, i.e. the Kanken, which a large number of Japanese take voluntarily or semi-voluntarily. The Kanken started in 1975 and provides twelve levels of difficulty from the easiest (Level 10) to the most difficult (Level 1, including Pre-2 and Pre-1). From the entire dataset, the present study focused mainly on Level 2 data (mastery of 2,136 daily-use kanji; 12th school year level) of 33,659 people (aged 9–106 years) in 2006, and 16,971 (aged 8–91) in 2016, who had simultaneously taken the exam at public test sites open to everyone. Additionally, to examine the replicability of the factor structures derived from the main data, we used an independent dataset, namely, Level 2 data from 2006 and 2016 from two data points per year, i.e. 12,050 people (aged 9–82 years) and 9,255 (aged 10–78) from 2006; and 9,141 (aged 10–81) and 2,671 (aged 11–78) in 2016. Each of these groups had taken the exam at non-public test sites (schools or public offices) on two dates in each year. Furthermore, we considered level Pre-2 data (daily-use of 1,940 kanji; 10–11th school year level) of 17,796 people (aged 8–97 years) in 2006, and 12,586 (aged 9–92) in 2016; Level 3 data (daily-use of 1,607 kanji; 9th grade level) of 15,769 people (aged 8–106) in 2006, and 12,470 (aged 8–91) in 2016, and Level 4 data (daily-use kanji of 1,322; 7–8th grade level) of 9,125 people (aged 7–86) in 2006 and 6,227 (aged 8–91) in 2016, who took the exam at public sites. The extremely small samples of preschool age children (6 years or younger) were excluded from the analysis.

The following characteristics of the Kanken support the methodological validity of using this dataset in the study: (1) as many as ten subtests that could broadly measure likely components of Japanese kanji abilities, which include reading and writing accuracy, and semantic comprehension; (2) simultaneous implementation at more than one public site in each of the 47 prefectures in Japan, thus reducing region-specific effects; (3) multiple levels of difficulty and multisite implementation around the same period using alternative versions of exam papers, which enabled us to examine the replicability of the results of factor analyses; (4) a large number of examinees varying in age from elementary school age to advanced age; and, (5) available data from 2006–2016. In this time period, the first smartphone, the iPhone, was released in 2007 and introduced in Japan in 2008. Smartphones have achieved widespread use in Japan. In 2016, their ownership ratio by age group was as follows: 99.4% by 20- to 29-year-olds; 96.2% by 30–39 years of age; 90.7% by 40–49; 86.5% by 50–59^20^.

All procedures in this study were approved by the Ethics Committee of the Unit for Advanced Study of Mind at Kyoto University, and were conducted in accordance with the Code of Ethics and Conduct of the Japanese Psychological Association. The data handled in this study was de-identified prior to being provided by the Japan Kanji Aptitude Testing Foundation.

### Measures

**Figure 1:**
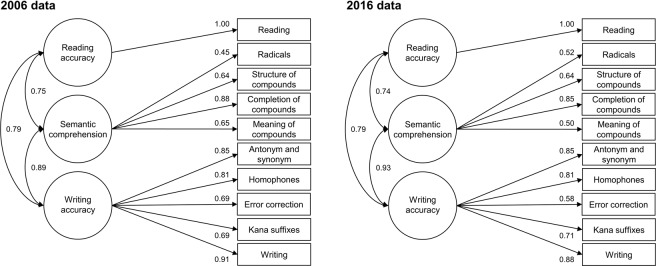
Illustration of the three-dimensional model of Japanese kanji abilities: the results of confirmatory factor analyses of the Level 2 data of the kanji exam implemented at public sites in 2006 (the left figure) and 2016 (the right one). *Numbers on single-headed arrows* indicate factor loadings. *Numbers on double-headed arrows* represent correlations among factors.

Level 2 of the Kanken is composed of ten subtests including (1) Reading, (2) Radicals, (3) Structure of compounds, (4) Completion of compounds, (5) Meaning of compounds, (6) Antonyms and synonyms, (7) Homophones, (8) Error correction, (9) Kana suffixes, and (10) Writing. Based on the nature of tasks, we hypothesized the correspondence between subtests and likely factors as follows: subtest 1 and reading accuracy, subtests 2–5 and semantic comprehension, subtests 6–10 and writing accuracy (see Fig. 1). The time limit for this level of the exam was 1 hour and criterion for certification was 80% or higher of a maximum score of 200. Pass rates for this level in 2016 were 19.1–23.1%.

Reading accuracy

1. Reading: This subtest requires examinees to write the correct pronunciation (i.e., convert it to kana) of a marked kanji word appearing in each of 30 sentences, taking context into consideration. Again, a kanji word can alternatively be written only with kana letters that have highly regular letter-sound correspondence. Thus, this conversion from kanji characters to kana letters is usually used as a kanji education in Japan. Each correct item was given a score of 1, adding up to a maximum of 30.

Semantic comprehension

2. Radicals: The examinees were required to extract a radical from each of 10 kanji characters. Radicals are visual components of kanji characters, most of which represent the semantic category, as the left part of the kanji 海 (*umi* or *kai*, sea) is regarded as a radical 氵 (*sanzui*) that means “water” or “fluid”. In general Japanese kanji dictionaries, 214 radicals are used for classifying kanji characters and each kanji is assigned one radical. Each correct item was given a score of 1, adding up to a maximum of 10.
3. Structure of compounds: This subtest requires examinees to classify 10 two-character kanji compounds into five categories based on their structure. The categories included cases where the two characters have similar meaning, two characters have opposite meaning, the former modify the latter, the latter is an object/complement of the former, and the former deny the meaning of the latter. Each correct item was given a score of 2, adding to a maximum of 20.
4. Completion of compounds: Examinees were required to complete 10 four-character kanji compounds by choosing one that precedes or follows each of 10 two-character kanji compounds from kana words and converting kana to kanji. There were ten prepared options of kana words. Each correct item was given a score of 2, adding up to a maximum of 20.
5. Meaning of compounds: This subtest requires examinees to choose one option that represents the meaning of 5 sentences from 10 four-character kanji compounds in subtest 4. Each correct item was given a score of 2, adding up to a maximum of 10.

Writing accuracy

6. Antonyms and synonyms: The examinees were required to choose an antonym or synonym for each of 10 two-character kanji compounds from kana words and write it correctly in kanji. There were ten prepared options of kana words. Each correct item was given a score of 2, adding up to a maximum of 20.
7. Homophones: This subtest required examinees to differentially write two homophones of kanji words that were written as marked kana letters in each of 5 pairs of sentences. Each correct item was given a score of 2, adding up to a maximum of 20.
8. Error correction: The examinees were required to identify a homophonic error of a kanji character in each of 5 sentences and write the correct one. Each correct item was given a score of 2, adding up to a maximum of 10.
9. Kana suffixes: This subtest required examinees to write a correct kanji character and a kana suffix accompanying it, based on marked kana letters in each of 5 sentences. Each correct item was given a score of 2, adding up to a maximum of 10.
10. Writing: The examinees were required to write a correct kanji word that was written as marked kana letters in each of 25 sentences. Each correct item was given a score of 2, adding up to a maximum of 50.

#### Subtests in the other levels

Whereas all subtests of the Level Pre-2 of the 2016 data coincided with those of Level 2, some alternatives were involved in the Level Pre-2 of the 2006 data and Levels 3 and 4 of both datasets. For the Level Pre-2 of the 2006 data, we hypothesized that semantic comprehension factors included Radicals, Structure of compounds, Completion of compounds (two subtests: one employed four-character compounds and another used two-character ones), and Homophones (all of the subtests excluding Radicals were multiple-choice questions). We hypothesized that writing accuracy factors included Antonyms and synonyms, Error correction, Kana suffixes, and Writing. For Levels 3 and 4 of both cohorts, we hypothesized that semantic factors included Radicals, Structure of compounds, Completion of compounds (two-character), and Homophones (the latter three employed multiple-choice), and writing factors included Antonyms and synonyms, Error correction, Kana suffixes, Writing, and Completion of compounds (a task that requires examinees to write a correct kanji character that was written as marked kana letters in each four-character compound). In all cases, the reading accuracy factor included only the Reading subtest.

### Statistical analyses

Data were analyzed in four steps. All statistical analyses were conducted using R version 3.4.3 (The R Foundation for Statistical Computing, Vienna, Austria).

Step 1: The goodness of fit for each of three structural models of Japanese kanji abilities was assessed with CFAs with the Level 2 data of the Kanken implemented at public sites (2006, 2016), using maximum likelihood estimation. In addition to the traditional χ^2^ statistics, the following indices of model fit were employed: the root mean square error of approximation (RMSEA) with its 90% confidence interval, the comparative fit index (CFI), the Tucker-Lewis index (TLI), the standardized root mean square residual (SRMR), and Akaike’s information criteria (AIC). RMSEA values <0.05 suggest a good fit, and values <0.08 are considered acceptable. We also calculated *p*-values for the test of the close-fit hypothesis that RMSEA ≤0.05. This one-sided null hypothesis should be adopted (i.e. *p* close ≥0.05) for a good fit^21^. The CFI and TLI values should be >0.95, the SRMR values should be <0.08 for a good fit, and lower AICs indicate relatively better fit^22–24^. Furthermore, internal consistency was assessed with the coefficient omega of composite reliability^25^ for the subtests loaded by each factor after these CFAs, as well as for all ten subtests before the analyses.

Step 2: To examine the replicability of the best fitting model identified in Step 1, CFAs were undertaken with the Level 2 data of the Kanken implemented at non-public sites on the two dates and the Level Pre-2, 3, and 4 data implemented at public sites in 2006 and 2016. Maximum likelihood was used for parameter estimation. Composite reliability was calculated for the subtests loaded by each factor.

Step 3: One-way analyses of variance (ANOVAs) were used to investigate factor-specific differences among four age groups, including high school (13–18 years), university (19–22 years), early (23–39 years) and middle adult (40–59 years). Children aged twelve or younger (*n* = 149, in 2006; *n* = 93, in 2016) and adults aged sixty or older (*n* = 652, in 2006; *n* = 908, in 2016) were excluded from the analyses in Steps 3 and 4, because of small sample sizes and concerns about the effects of cognitive decline caused by normal aging and neurodegenerative diseases in the case of the latter. These analyses employed the sums of standardized scores (*z*-scores) on subtests loaded by each factor in the comparable level 2 data of the exam implemented at public sites in 2006 and 2016, to examine the age effects on the abilities of each cohort. Tukey’s HSD tests were used for post-hoc comparisons among age groups. Statistical testing in steps 3 and 4 was two-tailed, and α was set at 0.05.

Step 4: Pearson’s correlation coefficients among factors were compared by age group with Fisher’s z test. These analyses also used the sums of *z*-scores on subtests loaded by each factor in the comparable level 2 data of the exam implemented at public sites in 2006 and 2016, to assess the age effects on the correlations in each cohort. To see the simple effects of the Kanken total scores on the correlations among factors, we compared the correlations in the two groups including the examinees who got median or higher total Kanken scores or the examinees who got lower scores.

## Results

Means and standard deviations of total and subtest scores on Level 2 of the Kanken implemented at public sites in 2006 and 2016 are shown in Table 1. As expected, raw scores on each subtest in these two datasets were broadly similar to each other, though it was hard to draw rigorous direct comparisons between the scores on the different test papers.

**Table 1:** Total and subtest scores on Level 2 of the kanji exam implemented at public test sites in 2006 and 2016.

	2006 data (*n* = 33,659) Mean (SD)	2016 data (*n* = 16,971) Mean (SD)
Reading accuracy		
Reading	25.61 (3.46)	25.17 (3.76)
Semantic comprehension		
Radicals	6.65 (1.78)	6.08 (1.85)
Structure of compounds	13.79 (3.98)	13.50 (3.87)
Completion of compounds	10.58 (5.39)	10.20 (5.14)
Meaning of compounds	7.91 (1.90)	8.22 (2.07)
Writing accuracy		
Antonyms and synonyms	12.93 (4.99)	12.02 (5.56)
Homophones	14.18 (4.15)	13.19 (3.97)
Error correction	6.51 (2.71)	6.21 (2.43)
Kana suffixes	6.48 (2.70)	6.44 (2.88)
Writing	33.31 (9.78)	34.09 (9.50)
Total Score	137.94 (32.59)	135.12 (32.23)

Composite reliability coefficients estimated for all ten subtests of these level 2 data in 2006 and 2016 were 0.94 and 0.93 respectively, and all item-total correlations without that item itself were 0.44 or higher (0.77, 0.44, 0.60, 0.78, 0.57, 0.82, 0.77, 0.66, 0.65, 0.85, in the 2006 data; 0.76, 0.48, 0.60, 0.77, 0.46, 0.81, 0.77, 0.56, 0.68, 0.83, in the 2016 data). These results suggest an acceptable level of internal consistency of the kanken as a measure of Japanese kanji ability.

### Goodness of fit for three models

We administered CFAs for the three-, two-, and unidimensional models with these level 2 data in 2006 and 2016, to examine the validity of multidimensional models of Japanese kanji abilities and the hypothetical relationships between subtests and possible factors. The results of CFAs are shown in Table 2. The three- and two-dimensional models were not nested. The two-dimensional model we hypothesized was composed of the reading comprehension factors (including subtests of Reading, Radicals, Structure of compounds, and Meaning of compounds), and the writing accuracy factors (including subtests of Completion of compounds, Antonyms and Synonyms, Homophones, Error correction, Kana suffixes, and Writing). In the latter group of subtests, examinees were required to write kanji characters accurately, whereas each item of the former ones did not require them to write a whole kanji character but rather kana letters, copy a part (radical) of kanji character, or fill in the bubble.

**Table 2:** CFAs for the three-, two-, and unidimensional models with Level 2 of the kanji exam implemented at public test sites in 2006 and 2016.

Data	χ^2^	*df*	*p*-value	RMSEA	[90%CI]	*p* close	CFI	TLI	SRMR	AIC
Level 2 in 2006 (*n* = 33,659)										
Three-dimensional	2530.834	33	<0.001	0.047	[0.046, 0.049]	0.997	0.987	0.983	0.022	1626158.390
Two-dimensional	5648.471	34	<0.001	0.070	[0.069, 0.072]	<0.001	0.971	0.962	0.028	1629274.027
Unidimensional	5874.673	35	<0.001	0.070	[0.069, 0.072]	<0.001	0.970	0.961	0.028	1629498.229
Level 2 in 2016 (*n* = 16,971)										
Three-dimensional	1698.293	33	<0.001	0.055	[0.052, 0.057]	<0.001	0.982	0.975	0.022	832142.732
Two-dimensional	2289.338	34	<0.001	0.063	[0.060, 0.065]	<0.001	0.975	0.967	0.025	832731.776
Unidimensional	2327.763	35	<0.001	0.062	[0.060, 0.064]	<0.001	0.975	0.967	0.026	832768.201

Note: CFA = confirmatory factor analysis, χ^2^ = chi-square statistic, *df* = degree of freedom of χ^2^ distribution, *p*-value = significance in χ^2^ test, RMSEA = root mean square error of approximation, 90%CI = 90% confidence interval of RMSEA, *p* close = *p*-value for the test of the close-fit hypothesis that RMSEA ≤ 0.05, CFI = comparative fit index, TLI = Tucker-Lewis index, SRMR = standardized root mean square residual, AIC = Akaike’s information criterion.

CFAs with Level 2 data from 2006 showed that the RMSEA estimate for the three-dimensional model (0.047) was lower than those for the two- (0.070) and unidimensional models (0.070), and tests for closeness of fit indicated significance only for the three-dimensional one (*p* close > 0.05). Additionally, the AIC value for the three dimensional model was lower than those for the other two. The CFI, TLI, and SRMR values indicated a good fit for all three models, though χ^2^ statistics were significantly large due to the large sample sizes. In the three-dimensional model (Fig. 1), composite reliability coefficients for semantic comprehension (0.81) and writing accuracy (0.92) were adequate, and those for reading comprehension (0.74) and writing accuracy (0.92) were also acceptable in the two-dimensional model.

In line with the results of CFAs with the 2006 data, the RMSEA for the three-dimensional model with the 2016 data (0.055) was also lower than those for the two- (0.063) and unidimensional ones (0.062), though all of them were not significant on statistical tests (all *p* close ≤ 0.05). Similarly, the lowest AIC value for the three dimensional model was replicated with the 2016 data, and the CFI, TLI, and SRMR values also indicated good fits for all three models. Composite reliability coefficients for semantic comprehension (0.78) and writing accuracy (0.90) in the three-dimensional model and for reading comprehension (0.72) and writing accuracy (0.92) in the two-dimensional model were at a similar level to those with the 2006 data.

### Replicability of model fit

To examine the replicability of the good fit for the three-dimensional model, we administered the CFAs with the Level 2 data of the exam implemented at non-public sites on two dates and the Level Pre-2, 3, and 4 data at public sites in 2006 and 2016. The results of the CFAs are shown in Table 3.

**Table 3:** CFAs for the three-dimensional model for Level 2 of the kanji exam implemented at non-public test sites and the Level Pre-2, 3, and 4 at public sites in 2006 and 2016.

Data	χ^2^	*df*	*p*-value	RMSEA	[90%CI]	*p*-value	CFI	TLI	SRMR	AIC
Level 2 at non-public site in 2006										
Earlier date (*n* = 12,050)	1529.351	33	<0.001	0.061	[0.059, 0.064]	<0.001	0.973	0.963	0.028	600208.736
Later date (*n* = 9,255)	569.301	33	<0.001	0.043	[0.040, 0.046]	1.000	0.987	0.983	0.022	450566.101
Level 2 at non-public site in 2016										
Earlier date (*n* = 9,141)	519.204	33	<0.001	0.040	[0.037, 0.043]	1.000	0.988	0.983	0.019	453343.586
Later date (*n* = 2,671)	287.603	33	<0.001	0.054	[0.048, 0.060]	0.134	0.979	0.972	0.023	132694.576
Level Pre-2 at public site										
2006 data (*n* = 17,796)	2976.545	33	<0.001	0.071	[0.069, 0.073]	<0.001	0.963	0.949	0.032	854549.924
2016 data (*n* = 12,586)	1859.823	33	<0.001	0.066	[0.064, 0.069]	<0.001	0.967	0.955	0.030	616708.464
Level 3 at public test site										
2006 data (*n* = 15,769)	2073.208	33	<0.001	0.063	[0.060, 0.065]	<0.001	0.973	0.963	0.028	764948.286
2016 data (*n* = 12,470)	1026.299	33	<0.001	0.049	[0.047, 0.052]	0.705	0.983	0.977	0.020	596419.704
Level 4 at public test site										
2006 data (*n* = 9,125)	1148.693	33	<0.001	0.061	[0.058, 0.064]	<0.001	0.983	0.977	0.037	438857.068
2016 data (*n* = 6,227)	732.797	33	<0.001	0.058	[0.055, 0.062]	<0.001	0.976	0.967	0.026	304545.170

Note: CFA = confirmatory factor analysis, χ^2^ = chi-square statistic, *df* = degree of freedom of χ^2^ distribution, *p*-value = significance in χ^2^ test, RMSEA = root mean square error of approximation, 90%CI = 90% confidence interval of RMSEA, *p* close = *p*-value for the test of the close-fit hypothesis that RMSEA ≤ 0.05, CFI = comparative fit index, TLI = Tucker-Lewis index, SRMR = standardized root mean square residual, AIC = Akaike’s information criterion.

The CFAs showed that the RMSEA estimated with the Level 2 data of the exam implemented at non-public sites at the later date in 2006 (0.43) and at both dates in 2016 (0.040, 0.054, respectively) and the Level 3 data at public sites in 2016 (0.049) were significantly low (all *p* close > 0.05), and acceptable in all other cases (all RMSEA ≤0.071). In addition, the CFI, TLI, and SRMR values with these data indicated a preferred fit for the three-dimensional model. Composite reliability coefficients with these data for writing accuracy were adequate (0.86 to 1.02). The coefficients for semantic comprehension were also acceptable (0.70 to 0.77) in most cases, though those with the Level 2 data of the exam implemented at the earlier date in 2016 (0.68), and the Level 3 (0.67) and Level 4 (0.68) of the 2016 data were slightly low.

### Effects of age on the scores in each cohort

We administered one-way ANOVAs for four age groups on the sums of *z*-scores in each of three dimensions of Japanese kanji abilities, using comparable level 2 data in 2006 and 2016, to examine the effects of age in each cohort. The results of ANOVAs are shown in Table 4.

**Table 4:** Composite scores of subtests for each factor by age group.

Age-group	Composite scores on each factor Mean (SD)			ANOVA (Multiple comparison: Tukey’s HSD)			Difference in Correlations
	Reading	Semantic	Writing	Reading	Semantic	Writing	SC and WA
Level 2 in 2006				*F* = 985.8***	*F* = 693.6***	*F* = 430.7***	
High school age (n = 10,969)	24.59 (3.60)	36.30 (9.43)	68.61 (20.52)	H. vs. U.***	***	***	*z* = 4.1***
University age (n = 10,745)	25.24 (3.42)	37.71 (10.04)	72.48 (20.60)	H. vs. E.***	***	***	*z* = 10.6***
Early adult (n = 6,882)	26.52 (3.16)	41.43 (10.64)	78.48 (21.17)	H. vs. M.***	***	***	*z* = 8.2***
Middle adult (n = 4,262)	27.43 (2.47)	43.52 (10.07)	79.32 (20.64)	U. vs. E.***	***	***	*z* = 7.0 ***
				U. vs. M.***	***	***	*z* = 5.1 ***
				E. vs. M.***	***		*z* = 0.8
Level 2 in 2016				*F* = 178.7***	*F* = 262.2***	*F* = 157.3***	
High school age (n = 6,556)	24.43 (3.91)	35.32 (9.44)	67.88 (20.02)	H. vs. U. ***	***	***	*z* = 2.5 *
University age (n = 4,421)	25.03 (3.72)	37.93 (9.65)	71.92 (20.33)	H. vs. E.***	***	***	*z* = 3.6***
Early adult (n = 2,290)	25.48 (3.73)	39.57 (10.15)	74.42 (21.62)	H. vs. M.***	***	***	*z* = 3.1***
Middle adult (n = 2,703)	26.33 (3.11)	41.12 (9.88)	76.83 (21.18)	U. vs. E.***	***	***	*z* = 1.6
				U. vs. M.***	***	***	*z* = 0.9
				E. vs. M.***	***	***	*z* = 0.6

* < 0.05, ** < 0.01, *** < 0.001.

Note: ANOVA = analysis of variance, SC = semantic comprehension, WA = writing accuracy, H = high school age (13–18 years), U = university age (19–22 years), E = early adult (23–39 years), M = middle adult (40–59 years).

The one-way ANOVAs with both the 2006 and 2016 data showed the main effects of age group on the scores in each dimension (*F*(3, 32854) = 985.8, *p* < 0.001, on reading accuracy, *F*(3, 32854) = 693.6, *p* < 0.001, semantic comprehension, *F*(3, 32854) = 430.7, *p* < 0.001, writing accuracy, in 2006; *F*(3, 15966) = 178.7, *p* < 0.001, reading accuracy, *F*(3, 15966) = 262.2, *p* < 0.001, semantic comprehension, *F*(3, 15966) = 157.3, *p* < 0.001, writing accuracy, in 2016). The post-hoc comparisons (Tukey’s HSD) revealed that the scores of university age examinees were higher than those of high-school age children, and the scores of young adults were higher than those of university and high-school age on all three dimensions (all *p* < 0.001). In addition, the scores of middle adults were higher than those of young adults and high-school and university age examinees on reading accuracy and semantic comprehension, in both 2006 and 2016 (all *p* < 0.001). In terms of writing accuracy, the scores for this dimension in middle adults were different from those of high-school and university age (all *p* < 0.001) but not young adults in 2006 (*p* = 0.660), whereas significant differences among these age groups were shown in 2016 (all *p* < 0.001).

### Effects of age on the correlations among dimensions in each cohort

To examine the effects of age on integration of kanji abilities in each cohort, we compared correlations among dimensions by age group (Table 4). We investigated only the correlations between the dimensions of semantic comprehension and writing accuracy, because of the ceiling effect of the Reading subtest. In Level 2 of the 2006 data, 72.8% of middle adult examinees and 60.3% of young adults got 90% or higher on the Reading subtest. Similarly, 56.7% of middle adults and 47.3% of young adults got 90% or higher on the subtest in 2016.

The correlations between the sums of *z*-scores on subtests loaded by semantic comprehension and writing accuracy factors in university age examinees (*r* = 0.73) and young (*r* = 0.77) and middle adults (*r* = 0.77) were higher than that of high-school children (*r* = 0.70) in 2006 (*z* = 4.1, *p* < 0.001, *z* = 10.6, *p* < 0.001, *z* = 8.2, *p* < 0.001, respectively). In addition, the significant differences between the correlations in high-school age (*r* = 0.71) and other groups (*r* = 0.73, *r* = 0.75, *r* = 0.74, respectively) were shown also in 2016 (*z* = 2.5, *p* = 0.01, *z* = 3.6, *p* < 0.001, *z* = 3.1, *p* < 0.001, respectively). In contrast, the correlations in young and middle adults were not higher than that of university age examinees in 2016 (*z* = 1.6, *p* = 0.12, *z* = 0.9, *p* = 0.37, respectively), whereas the differences between those correlations were also shown in 2006 (*z* = 7.0, *p* < 0.001, *z* = 5.1, *p* < 0.001, respectively). The correlations in middle adults were not higher than those of young adults in both 2006 (*z* = 0.8, *p* = 0.45) and 2016 cohorts (*z* = 0.6, *p* = 0.52). In contrast to the higher correlations in the older groups whose scores were generally higher, supplementary analyses showed that the correlations in the high score group (*r* = 0.43, *n* = 16,441) were not higher than the low score group (*r* = 0.42, *n* = 16,417) in 2006 (*z* = 1.43, *p* = 0.15). In the 2016 data, the correlations in the examinees with high scores (*r* = 0.38, *n* = 8,077) were lower than that of people with low scores (*r* = 0.44, *n* = 7,893; *z* = 4.62, *p* < 0.001).

## Discussion

This study of the most popular kanji exam in Japan tested three hypotheses concerning the dimensionality of Japanese kanji abilities and age effects on them in two cohorts ten years apart. Our results supported (1) the three-dimensional model of Japanese kanji abilities, including facets of reading, writing, and semantic comprehension (see Fig. 1); (2) showed that each ability, as well as the correlations between them, grew with increasing age from adolescence to adulthood, however, (3) the pattern of age-related increase in the writing and orthography-semantics relationship are different for 2006 and 2016. These findings represent the first reported evidence of the multidimensional factor structure of Japanese kanji abilities, and the age/cohort differences between them.

### Multidimensionality of Japanese kanji abilities

The good fit of the three-dimensional model for this large dataset supports the supposition that Japanese kanji abilities largely depend on the lexical route in dual-route models. It is assumed that multiple abilities reflecting phonological, orthographic, or semantic processing are needed to master and manage kanji characters that have multiple pronunciations, meaning and visual complexity.

The underlying basis or cognitive demands of literacy acquisition include not only universal but language-specific factors^26^. Before now, visual memory^27^, visuospatial cognition^3^, and morphological awareness^28^ have been reported as cognitive predictors of Japanese kanji acquisition, in addition to relatively small contributions of phonological processing and rapid automatized naming, crucial in alphabetical orthographies^29^. These reports suggest a broad range of cognitive functions underpin multidimensional kanji abilities in Japanese. Decades ago, low prevalence of developmental dyslexia was reported in Japan (0.1–2%)^30,31^. However, as noted by Uno and coauthors^3^, many more people than previously recognized may experience difficulties in kanji acquisition caused by a variety of cognitive atypicalities.

### Different effects of age in the 2006 and 2016 cohorts

Different patterns of age effects were observed in the two cohorts. First, whereas writing accuracy reached a peak in early adulthood in 2006, a further increase in this area was observed from early to middle adulthood in 2016. Second, whereas the correlations between writing accuracy and semantic comprehension also peaked in early adulthood in 2006, an increase in these areas was not observed after university age in 2016. These results indicate reduced kanji writing ability in young adults, as well as weakened orthographic-semantics relationships in adults, a decade later. A possible explanation for this is that the rapid spread of digital writing devices, such as PCs and smartphones^20^ that provide the correct kanji or at least options from which to select them, have affected kanji writing ability in the past decade. Alternatively, or in addition, increased attention to learning English^18^ or internationalization, which may have resulted in a decrease in the time and effort dedicated to learning kanji, could affect kanji skills.

Although the strong correlations between writing and semantic factors appear to be contrary to the distinctiveness of the dimensions, these close relationships are expected considering the highly consistent relationship between kanji orthography and semantics^32^. Even when the meaning of kanji is unknown, we can sometimes read them aloud. However, in such cases, we cannot single out the correct kanji character from a number of homophones. A decreased orthographic-semantics relationship suggests increased ambiguity in the differential use of homophonic kanji in current-day Japanese adults, which could lead to reduction in use frequency of specific kanji characters or possibly kanji itself.

Finally, our results imply that the habit of handwriting, once essential for daily tasks, may be advantageous for the acquisition of writing-related skills in Japanese. In the present day, digital writing devices are increasingly replacing handwriting not only in Japan but also worldwide, however, whether this technology should be applied to early literacy education is controversial^33,34^. On the one hand, the ease of typing or other supportive capabilities of digital tools are seen to be advantageous for literacy learning, particularly in children with undeveloped motor skills^35^ or reading/writing difficulties^36^. On the other hand, it has been argued that the coupling of motor action and perception during handwriting can facilitate literacy acquisition, based on evidence from experimental^37^, neuroimaging^38^, and intervention studies^39^. Our data support the latter view and imply that keeping the habit of handwriting may be important for integrated mastery of higher-level literacy skills in adults.

### Limitations

First, in this retrospective analyses of data obtained not for research, the sample was not randomly extracted from each age group of the general population. Differences in motivation for taking the exam may have affected scores. Second, this cross-sectional study cannot indicate whether the observed age-related differences reflect a developmental process. Finally, the ceiling effects of the Reading subtest possibly influenced the results of the CFAs. The distinctiveness of the reading dimension or the relationships with the other two dimensions need to be examined in future.

## Conclusion

The current study showed new evidence of the multidimensional nature of Japanese kanji abilities composed of reading, writing, and semantic comprehension. The different pattern of age-related effects on the abilities between 2006 and 2016 cohorts suggested reduced kanji writing ability and stagnation in integrated mastery of kanji orthography and semantics in current-day Japanese adults. This decline appears against the backdrop of the rapidly spreading use of digital writing devices and/or increased attention to learning English or internationalization. These findings warrant further research on cognitive and neurobiological bases of kanji acquisition in Japanese people, and the effect of handwriting on literacy acquisition in children and adults acquiring Japanese or other orthographies.

## Data Availability

The data analyzed in this study is available from the corresponding author upon reasonable request.
